# Some Notes Bearing on the Administration of Iron

**Published:** 1891-03

**Authors:** John Aulde

**Affiliations:** Philadelphia, Pa.


### SOME NOTES BEARING ON THE ADMINISTRATION OF IRON.

By JOHN AULDE, M. D., Philadelphia, Pa.

ALTHOUGH iron is highly esteemed as a medicament, and is largely used for its tonic effect upon the system, so frequently does it occur that the patient objects, owing to some idiosyncrasy or fancy, that we cannot regard it wholly as an ideal hematinic. No apology, therefore, is required in offering to the profession a comparatively recent preparation, which is free from some of the objections that have been urged against many of the iron preparations now in use. In order to make the reasons which I have to offer clear and distinct to the casual reader, I have deemed it wise to consider briefly some points intimately connected with the pharmacology of the drug. From this preliminary study we shall be in a measure prepared to estimate how nearly the new product comes to meeting the defects with which we have had to contend so long, and at the same time it may possibly lead to a more intelligent use of this well-known remedy.

Besides the reduced iron, we have in general use the ferric and ferrous preparations, the latter being more mild, less astringent, and free from the objections to the ferric salts — that of coagulating albumin. Lethal doses of the ferric salts used intravenously, in experimental investigations, cause almost immediate paralysis of the central nervous system, fall of blood-pressure, and death. Although the perchloride, when thus used, causes instant death by coagulation of the blood, it does not act in this direct manner when introduced subcutaneously; the nerves are unaffected, but at the points of elimination inflammatory action is set up, *e. g.*, the kidneys, liver, and intestinal mucous membrane show more or less effect.

Absorption takes place as a peptonate or albuminate, but it is taken up so slowly that no appreciable result follows, unless, as just stated, it may be used intravenously or subcutaneously. Absorption takes place more rapidly in catarrhal conditions of the intestinal tract — a fact to be borne in mind when exhibiting large doses, which cause gastro-intestinal catarrh. Small doses do not have this effect, nor does the metal appear in the urine from their administration, such as may be observed after the ingestion of large doses. It will be inferred from the foregoing that by the exhibition of small doses of a soluble preparation of iron it will be assimilated without causing derangement of the alimentary tract, and in this way the secondary effects, *i. e.*, the deposit of the metal in the system, may be avoided.

The fact should be kept constantly in view, that metals have a poisonous action upon nerves, nerve-centres, muscles, and upon all glandular structures; and as iron is a reputed hematinic, much harm may result from its injudicious employment, as there are evidently certain toxic effects following the long-continued use of insoluble preparations. This is a rule which applies especially to all insoluble iron preparations, and it is but reasonable to assume that, whatever harm has been done through this means, may have escaped attention, because few physicians are likely to investigate the presence of factitious diseases. Another factor which has contributed to lessen these evils, is the slow process of absorption.

The foregoing observations apply with equal force to the effects of the drug upon the circulatory apparatus. While copper is an active agent in causing contraction of the blood-vessels, iron produces slow contractions, showing that it is less irritant (stimulant) to the nervous system. This may possibly be accounted for on the hypothesis that iron is a normal constituent of the blood. Whether this effect is due to irritation (stimulation) of the vaso-motor nerves, central or peripheral, or to a direct action upon the muscular walls of the blood-vessels, is a question still in doubt. My own impression is, that through the influence of the medicament upon the nerve-cells the large doses, comparatively, arrest their function, when contraction of the muscular structures in the vessels takes place. The ferric salts, owing to their property of coagulating albumin and blood, of course, produce more marked effects than the ferrous salts. Digitalis and ergot among the organic, and barium chloride among the inorganic, remedies, well-known as vascular tonics, furnish apt illustrations of this important principle.

Iron has a tendency to accumulate in the liver; small doses do not show this tendency, but they may serve to increase the functional activity of this organ, when given in a soluble, non-astringent form, by restoring cell-nutrition to the normal.

The effect of iron upon muscular structure has long been known to experimental physiologists, but I doubt if this knowledge is appreciated by many practitioners, who regard the possible benefits to be derived from the exhibition of iron preparations in proportion to the amount tolerated by the patient. Now, large doses, while they do not affect the irritability of muscular structure, lessen materially the amount of work it is capable of performing, while small doses increase the capacity of muscle for work. What is most to be desired, therefore, is a preparation not open to the objections inferred from these investigations; but owing to the necessity for consulting the palate of our patients, it is also desirable that the substance should be free from the nauseating effects which are so common to all preparations of iron. The combination, I believe, is to be found in that form known as levulose ferride, which was highly recommended to me several years ago by my friend, Dr. James Collins, of this city.

The preparation known as levulose ferride is one which takes the place of a well-known and popular German product, called *Eisenzucker* (iron sugar), very extensively used in domestic practice. I was led to the employment of iron-sugar on account of its palatability, fastidious patients and children making no objections to it; but this has been supplanted by levulose ferride, which in the form of tablet triturates will be taken as readily as chocolate bon-bons. It is readily soluble in an excess of water, and practically free from any ferruginous taste or styptic effect when dissolved in the mouth, and is substantially a peptonate. The method of preparing it is briefly as follows: To a certain amount of iron a measured quantity of malt-sugar (maltose) is added, and the mixture constantly stirred while exposed on a water-bath. While it possesses all the desirable qualities mentioned, the presence of metallic iron may be determined by chemical analysis, the strength of the product being about three per cent.

This preparation, it will be apparent, will act much less actively as an astringent than even the ferrous preparations; but, of course, it cannot be expected to take the place of the ferric products, which are sometimes demanded, as in the case of intestinal parasites (sarcina ventriculi and lumbricoides). On the other hand, it will be especially indicated for the relief of anemia and chlorosis, owing to its ready absorption, lack of astringency, and its palatability. In all cases of defective nutrition, from any cause, where the ingestion of any form of medicament is a trial to the patient, this product will be kindly received. A synopsis of some of the cases in which it is indicated, together with a summary of the effects following its employment, may prove interesting to the physician.

During the early Summer months, I had under observation a young mother with a six-months' old child, who presented a very anemic condition. I had seen her but once since the delivery of her child, and anticipating that she would not be able to nourish it sufficiently and maintain her health, I had cautioned her in regard to the most appropriate diet. Notwithstanding every care had been used, she was finally compelled to seek medical aid, or go to bed. All that this patient required was something for the purpose of increasing the amount of hemoglobin, which would restore the integrity of the red corpuscles and improve the oxygen-carrying capacity of the blood. This being most readily accomplished by levulose ferride, she was ordered to take tablets of this preparation, each containing three grains, after meals. To meet the emergency, and increase the patient's strength, until such time as the advantages of the iron would be apparent, small doses of strychnine (one-sixtieth grain) were administered along with the iron. Ordinarily, this class of patients, when they begin in the early Summer, suffer more or less from the effects of heat, and become regular patrons of the doctor; but this patient did not make her appearance again for about two months, when she said she thought it was about time to have a little more of the same medicine. I may mention in passing, that the first medicine was sufficient only to cover the first ten days, and the patient seemed greatly disappointed that she was compelled to return.

So many children are so promptly benefited by the use of a small quantity of iron, that it is a great drawback to us that no palatable preparation has been discovered and put on the market. I have in mind a little fellow, who has long been very much adverse to eating meat, due, I presume, to defective digestion; but for the past few weeks, since he has been taking the levulose ferride, he seems quite content to eat meat alone, and is becoming strong and robust. Not long ago I had a visit from a lady, who brought with her a young lad, aged fourteen, who had a most forbidding cadaveric expression, and he could eat no meat. His brother, I was told, had died about his age from Bright's disease, and this one presented all the symptoms peculiar to the brother who died. Still, with attention to diet, outdoor exercise in the country, and a tablet triturate containing three grains of levulose ferride after meals, he made a prompt recovery. Although I was unable to discover any symptoms of Bright's in this instance, I was impressed with the depression due to the anemic condition; and yet, without some readily assimilable iron preparation, it would have been a tedious process to start him on the way toward recovery.

Late in the Spring of the year, a gentleman, aged about thirty-five, called on me, complaining of dyspepsia, although he had been under the treatment of another physician for overwork for the preceding four years. After regulating his diet, and adopting treatment calculated to restore the activity of the digestive apparatus, he was placed upon levulose ferride along with strychnine sulphate — three grains of the former in tablet form, and one-sixtieth grain of the latter, and did remarkably well on this combination. This product, like all other mild preparations of iron, is mostly indicated in cases of this class, and along with these may be mentioned chorea, convalescence from lingering diseases, like typhoid fever; and in all such instances, I venture to anticipate that the results will be especially favorable where proper attention is given to the dietetic measures.

The administration of the remedy may be confined to the use of the powder, which is taken dry on the tongue, dissolved in water or coffee; or it will be found more convenient in the form of tablets, each containing three or five grains. The dose for children ranges from three to ten grains, and for adults from five to thirty grains.

The Levulose Ferride was obtained through Messrs. Eisner & Mendelson Co., of New York, who import this article.—*New England Medical Monthly.*

---

A Chinese Hospital in Brooklyn.—The articles of incorporation have been filed at Albany for a Chinese Hospital Association, to be located at Brooklyn. Clergymen and physicians of that city chiefly constitute the board of directors. It will be exclusively for the use of Chinamen, and it is not expected to receive contagious cases.—*St. Louis Medical and Surgical Journal.*

---

"I move to lay it on the table," said one of the medical students on their return from a "lifting" expedition; and the subject was promptly "tabled."—*St. Joseph Medical Herald.*

---

Quiz: — Student enumerating some of the local edemas: Hydrothorax, hydrocephalus, hydrocele — hydrophobia. Class applauds.

---

Picrotoxin in doses of one-sixtieth of a grain is excellent in checking the exhausting night sweats of phthisis.

